# Dynamic changes of serum soluble triggering receptor expressed on myeloid cells-1 (sTREM-1) reflect sepsis severity and can predict prognosis: a prospective study

**DOI:** 10.1186/1471-2334-11-53

**Published:** 2011-03-01

**Authors:** Jie Zhang, Danyang She, Dan Feng, Yanhong Jia, Lixin Xie

**Affiliations:** 1 Department of Respiratory Medicine, Chinese PLA General Hospital, 28 Fuxing Road, Beijing, China; 2 Department of Medical Statistics, Chinese PLA General Hospital, 28 Fuxing Road, Beijing, China; 3 a postgraduate of Medical College, Nankai University, Tianjin, China

## Abstract

**Background:**

We examined the utility of serum levels of soluble triggering receptor expressed on myeloid cells-1 (sTREM-1) for the diagnoses, severity assessments, and predicting the prognoses of patients with sepsis and compared sTREM-1 values with those of C-reactive protein (CRP) and procalcitonin (PCT).

**Methods:**

Fifty-two patients with sepsis were included: 15 sepsis cases and 37 severe sepsis cases (severe sepsis + septic shock). Serum levels of sTREM-1, CRP, and PCT were determined on days 1, 3, 5, 7, 10, and 14 after admission to an ICU.

**Results:**

Serum sTREM-1 levels of patients with severe sepsis were significantly higher than for those with sepsis on day 1 (240.6 pg/ml vs. 118.3 pg/ml; *P *< 0.01), but CRP and PCT levels were not significantly different between the two groups. The area under an ROC curve for sTREM-1 for severe sepsis patients was 0.823 (95% confidence interval: 0.690-0.957). Using 222.5 pg/ml of sTREM-1 as the cut-off value, the sensitivity was 59.5%, the specificity was 93.3%, the positive predictive value was 95.6%, the negative predictive value was 48.3%, the positive likelihood ratio was 8.92, and the negative likelihood ratio was 0.434. Based on 28-day survivals, sTREM-1 levels in the surviving group showed a tendency to decrease over time, while they tended to gradually increase in the non-surviving group. sTREM-1 levels in the non-surviving group were higher than those in the surviving group at all time points, whereas CRP and PCT levels showed a tendency to decrease over time in both groups. sTREM-1 levels and Sequential Organ Failure Assessment (SOFA) scores were positively correlated (r = 0.443; *P *< 0.001), and this correlation coefficient was greater than the correlation coefficients for both CRP and PCT.

**Conclusions:**

Serum sTREM-1 levels reflected the severity of sepsis more accurately than those of CRP and PCT and were more sensitive for dynamic evaluations of sepsis prognosis.

**Trial Registration:**

Current controlled trials ChiCTR-OCH-09000745

## Background

Sepsis is the most important cause of morbidity and mortality in the intensive care unit; however, sepsis lacks specific clinical manifestations. Thus, it is highly desirable to find sensitive and specific indicators of infection that can be easily collected, that accurately reflect infection severity and prognosis and are clinically important. Current common clinical indicators of infection include pyrexia, white blood cell counts, C-reactive protein (CRP) and procalcitonin (PCT).

Triggering receptor expressed on myeloid cells-1 (TREM-1), discovered by Bouchon et al. in 2000 [1], is a member of the immunoglobulin superfamily of receptors that is specifically expressed on the surfaces of monocytes and neutrophils. TREM-1 expression is increased in infectious diseases and is associated with the release of soluble TREM-1 (sTREM-1). One study by Gibot et al. [2] demonstrated that the value of plasma sTREM-1 levels as an indicator of sepsis was superior to CRP and PCT, although other studies reported that the value of sTREM-1 for diagnosing sepsis was inferior to CRP and PCT [3-5].

The purpose of this study was to track changes in serum sTREM-1, CRP and PCT levels in patients with sepsis and to compare the predictive values of these three factors for assessing sepsis and establishing prognosis.

## Methods

### Subjects

Between September 2009 and March 2010, inpatients were included who were in the intensive care units (ICU) of the Department of Respiratory Disease, the Emergency Department, and the Department of Surgery of the Chinese People's Liberation Army General Hospital. These patients were diagnosed with sepsis, severe sepsis, or septic shock according to the 1991 ACCP/SCCM Joint Meeting [6] and by the diagnostic criteria developed at the 2001 International Sepsis Definition Conference [7]. Patients were excluded if they were < 18 years old, died within 24 hours of admission, had neutropenia (< 500 neutrophils/mm^3^), had an acquired immunodeficiency syndrome, or refused to participate in this study.

Patients were divided into a sepsis group and a severe sepsis group (severe sepsis + septic shock), and additional analysis was based on 28-day survivals for a surviving group (≥ 28 days survival) and those who died (< 28 days survival). Patients or their family members were fully informed and signed informed consent forms. This study was approved by the Ethics Committee of the Chinese PLA General Hospital (project number 20090923-001).

### Data collection

Demographic and disease data of patients included age, gender, chief complaint for admission, vital signs, routine blood test results, liver and kidney functions, coagulation indicators, Acute Physiologic Assessment and Chronic Health Evaluation (APACHE) II scores, and Sequential Organ Failure Assessment (SOFA) scores. These were recorded on days 1, 3, 5, 7, 10, and 14. Serum was collected at these same time points and sTREM-1, CRP and PCT levels were determined.

### Assays

sTREM-1 was determined using a double antibody sandwich ELISA (Quantikine Human TREM-1 Immunoassay ELISA Kit, R & D Systems, Minneapolis, MN, USA, product number DTRM10B). CRP was determined using scattering turbidimetry (CardioPhase hsCRP, Siemens, Germany) and PCT was measured using an enzyme-linked fluorescence analysis kit (ELFA, VIDAS BRAHMS PCT kit, bioMerieux SA, France). All assays were performed according to the manufacturer's instructions.

### Statistical analysis

Quantitative data with normal distributions, including age, APACHE II scores, body temperature, and white blood cell counts (WBC), are given as means ± standard deviations (SD). Student's t-test was used to compare means between two groups. Quantitative data that were not normally distributed, including sTREM-1, CRP, PCT and SOFA scores, were summarized as medians (interquartile ranges) and compared by non-parametric tests (Mann-Whitney U test). Proportions were used to express qualitative data and the differences in proportions between groups were compared using a chi-square test. Spearman correlation coefficients were used to assess associations between SOFA scores and sTREM-1, CRP and PCT levels. Statistical analysis used SPSS Statistics 17.0.

## Results

### Patient Characteristics

Fifty-two patients with sepsis were included in this study. There were 15 cases with sepsis and 37 cases with severe sepsis. Thirty-one patients were male (59.6%) and the mean patient age was 56 ± 19 years. Initial sites of infection were the lungs (50%), blood (23%), and abdomen (13%). Patients' ages and day 1 temperatures were not significantly different between the two groups (*P *> 0.05). However, the APACHE II scores and white blood cell counts in the severe sepsis group were higher than those of the sepsis group (*P *= 0.006 and *P *= 0.003, respectively).

### Comparisons of initial sTREM-1, CRP and PCT levels

Serum sTREM-1 levels of patients in the severe sepsis group were significantly higher than those in the sepsis group on day 1 (240.6 pg/ml vs. 118.3 pg/ml, *P *< 0.01), but there were no significant differences in CRP or PCT levels between the two groups. SOFA scores of the severe sepsis group were significantly higher than those of the sepsis group (6 vs. 3; *P *< 0.001; Table 1).

**Table 1:** Serum sTREM-1, CRP, PCT levels and SOFA scores on day of enrollment.

Parameter	Sepsis (n = 15)	Severe sepsis (n = 37)	*P *value
sTREM-1 (pg/ml)	118.3 (124.5)	240.6 (180.2)	< 0.001
CRP (mg/dl)	10.60 (11.50)	11.70 (12.95)	0.920
PCT (ng/ml)	0.21 (2.50)	1.31 (15.03)	0.132
SOFA score	3 (4)	6 (6)	< 0.001

*: Data are presented as median (interquartile range).

**Figure 1:**
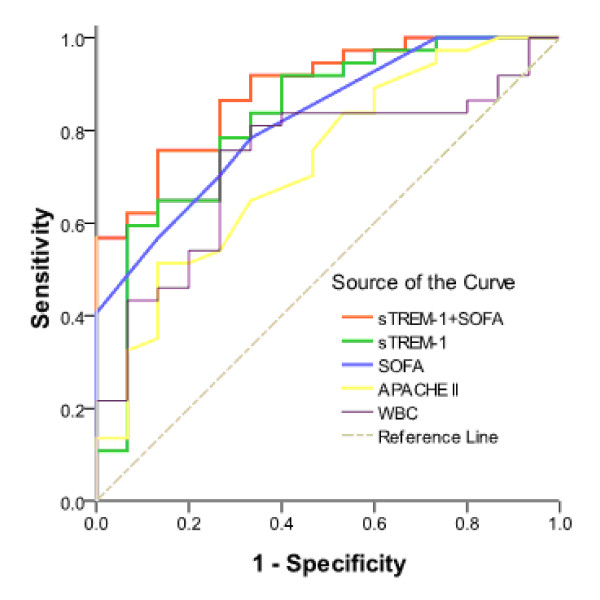
**ROC curves for APACHE II scores, WBCs, SOFA scores and sTREM-1 levels for distinguishing severe sepsis from sepsis**.

ROC curves for APACHE II scores, WBCs, SOFA scores and sTREM-1 levels for the two groups were constructed based on statistically significant differences (Figure 1) for calculating the areas under the ROC curves (Table 2). These results showed that combining both sTREM-1 and SOFA scores was better than any single indicator for diagnosing severe sepsis. We used the maximum Youden Index (YI) to select cut-off criteria values. Using a cut-off value for sTREM-1 of 222.5 pg/ml, the sensitivity was 59.5%, the specificity was 93.3%, the positive predictive value was 95.6%, the negative predictive value was 48.3%, the positive likelihood ratio was 8.92 and the negative likelihood ratio was 0.434 for severe sepsis diagnosis. Using a cut-off value for SOFA scores of 3.5, the sensitivity was 78.4% and the specificity was 66.7% for severe sepsis diagnosis.

**Table 2:** Area under ROC curve on diagnosing severe sepsis.

Parameters	AUC	Standard error (SE)	P value	95% confidence interval	
				
				Lower limit	Upper limit
sTREM-1	0.823	0.068	0.000	0.690	0.957
SOFA	0.823	0.060	0.000	0.706	0.939
sTREM-1+ SOFA	0.886	0.047	0.000	0.795	0.978
APACHE II score	0.726	0.078	0.011	0.574	0.879
WBC	0.735	0.074	0.008	0.590	0.880

### Dynamic changes of sTREM-1, CRP and PCT levels

To assess dynamic changes in serum levels of serum sTREM-1, CRP and PCT levels, patients were divided into either a surviving group (≥ 28 days survival) or a non-surviving group (< 28 days survival) based on 28-day survivals (Table 3). The number of patients in the non-surviving group decreased with increasing mortality. Thus, the actual numbers of patients in the non-surviving group were 16, 16, 14, 14, 12, and 9 on days 1, 3, 5, 7, 10, and 14, respectively. For patients who died within 14 days of admission, the last measurements obtained before these patients died were used for each of the time points after a patient's death.

**Table 3:** Comparison of patient demographics and clinical data between the survival group (≥ 28 days) and the non-survival group (< 28 days) based on 28-day survival.

Clinical features	Survival group (n = 36)	Non-survival group (n = 16)	P-value
Age (years)	59 ± 18	51 ± 22	NS
Male/Female	22/14	9/7	NS
Initial WBC (10^9/L)	12.4 ± 5.9	14.1 ± 5.9	NS
Initial APACHE II score	14.9 ± 7.1	19.7 ± 7.3	0.03
Initial SOFA score	4.0 (4.75)	9.5 (5.75)	0.001
sTREM-1 (pg/ml)	193.4 (179.9)	240.2 (257.5)	NS
CRP (mg/dl)	10.85 (11.48)	11.95 (12.20)	NS
PCT (ng/ml)	0.68 (10.93)	1.33 (12.26)	NS

*: Data are presented as mean ± standard deviation, number, or median (interquartile range).

**Figure 2:**
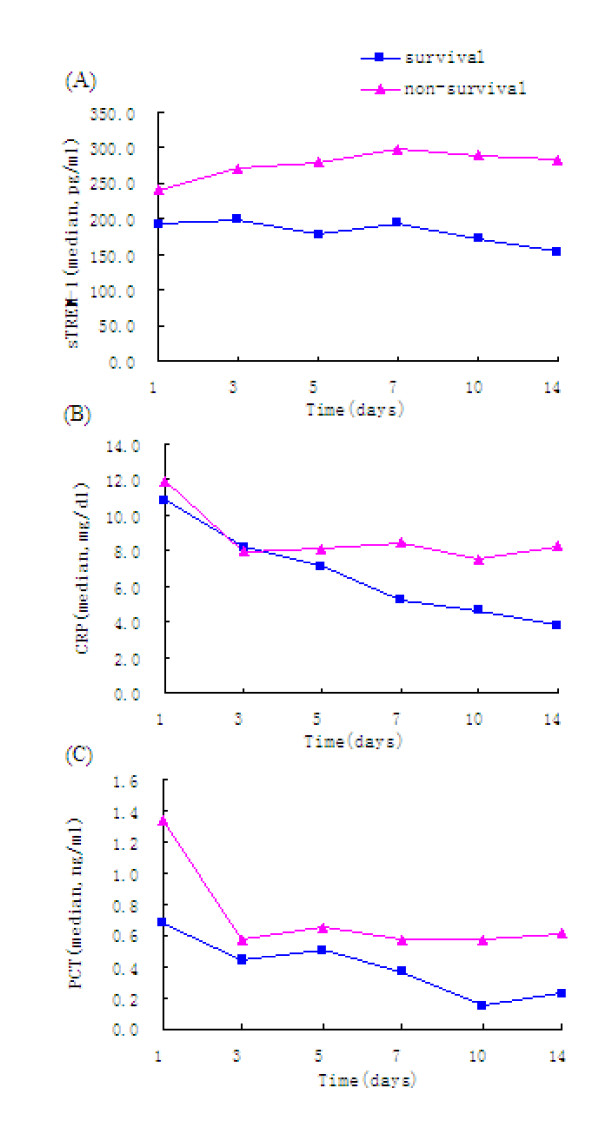
**Serum levels of (A) sTREM-1, (B) CRP, and (C) PCT measured over 14 days in patients diagnosed with sepsis based on 28-day survival**.

Median serum sTREM-1, CRP and PCT levels determined on days 1, 3, 5, 7, 10, and 14, and were compared between the surviving and non-surviving groups (Figure 2). Serum sTREM-1, CRP and PCT levels in the non-surviving group tended to be higher than those in the surviving group. In particular, serum sTREM-1, CRP and PCT levels in the non-surviving group were higher than those in the surviving group on days 1, 3, 5, and 7, although the differences were not statistically significant between the two groups (*P *> 0.05). Serum sTREM-1, CRP and PCT levels in the non-surviving group were, however, significantly higher than those in the surviving group on days 10 and 14 (*P *< 0.05).

Longitudinally, serum sTREM-1, CRP and PCT levels in the surviving group showed a tendency to decrease over time (*P *< 0.001). In the non-surviving group, the sTREM-1 levels tended to gradually increase with time, but the changes in sTREM-1 levels were not statistically significant (*P *= 0.222). In contrast, CRP and PCT levels tended to decrease over time, especially within the first 3 days.

Patients with higher serum levels of sTREM-1, CRP and PCT had poorer prognoses. Compared with the tendency for changing levels of CRP and PCT, the gradual increase of sTREM-1 levels was a better reflection of the progression of sepsis, which was of greater value for predicting death.

### Associations between SOFA scores and sTREM-1, CRP and PCT levels

**Figure 3:**
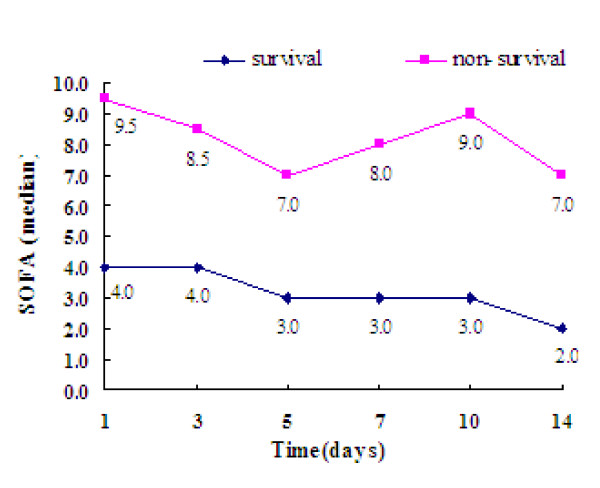
**Comparison of SOFA scores between patients in the surviving and non-surviving groups**.

SOFA scores in the surviving group were lower than those in the non-surviving group on day 1 (4.0 vs. 9.5; Z = -3.387; *P *= 0.001). SOFA scores in the surviving group gradually decreased as the course of the disease progressed. There was no apparent decrease in SOFA scores for the non-surviving group. Rather, SOFA scores in the non-surviving group showed a tendency to increase during the last days (Figure 3), suggesting that SOFA scores were closely related to the severity and prognosis of sepsis.

**Figure 4:**
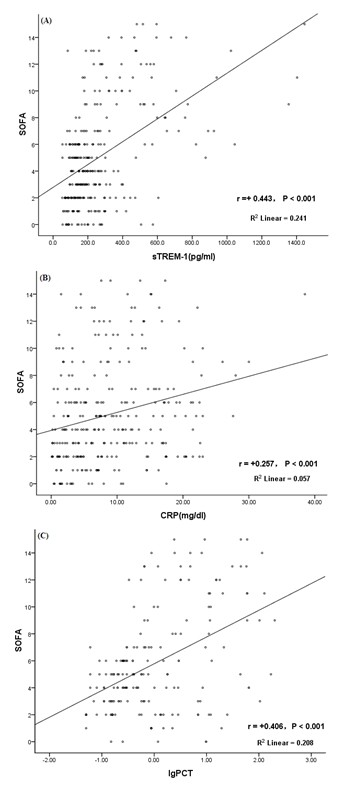
**Correlation between SOFA scores and serum (A) sTREM-1, (B) CRP, and (C) PCT levels**. The Spearman correlation coefficients (r) were 0.443, 0.257, and 0.406 (*P *< 0.001) between SOFA and serum sTREM-1, CRP, and PCT respectively (the logarithmic value of PCT was used for analysis).

As shown in Figure 4 Spearman correlation analysis was used evaluate the associations between SOFA scores and serum sTREM-1, CRP and PCT (logarithmic values of PCT were used for this analysis). The correlation coefficients (r) were 0.443, 0.257, and 0.406, respectively (*P *< 0.001).

## Discussion

During the course of sepsis, TREM-1 amplifies infection-induced inflammatory response signals primarily through the mediation of adapter protein DAP12 on the cell surface. sTREM-1 is the soluble form of TREM-1 that lacks the transmembrane and intracellular domains. These two domains are cleaved from TREM-1 on the membrane surface by proteolysis [8]. In 2009, a meta-analysis [9] found that the sensitivity of sTREM-1 for diagnosing bacterial infection was 0.82 (95% confidence interval [CI]: 0.68-0.90) and the specificity was 0.86 (95% CI: 0.77-0.91). These high values suggested that sTREM-1 was a reasonably reliable indicator for diagnosing bacterial infections.

Compared with previous studies, our study found that sTREM-1 was a valuable tool for diagnosing severe sepsis (severe sepsis + septic shock) with organ dysfunction. sTREM-1 levels in the severe sepsis group were significantly higher than those in the sepsis group starting at day 1 of ICU admission. Thus, sTREM-1 measurements may be useful for an early diagnosis of severe sepsis and timely intervention. There were no significant differences for either CRP or PCT levels between the sepsis and severe sepsis groups, implying that serum sTREM-1 was superior to CRP and PCT for diagnosing severe sepsis.

In our study, the sensitivity and specificity of sTREM-1 for diagnosing severe sepsis were 59.5% and 93.3%, respectively. The low sensitivity value may have been related to the small sample size, and follow-up studies should include larger samples. SOFA scores had a slightly higher sensitivity with a lower specificity. Therefore, combining both sTREM-1 levels and SOFA scores may be more valuable for diagnosing severe sepsis compared with any single indicator.

In our study, patients with more severe conditions had higher SOFA scores. Additionally, SOFA scores in the non-surviving group tended to increase over time, while they decreased gradually in the surviving group. Thus, SOFA scores can dynamically reflect an improvement in a patient's condition, or even the progression of sepsis. Consistent with the results of Dimopoulou et al. [10], we also found that serum sTREM-1 levels were positively correlated with the SOFA scores. Compared with CRP and PCT, sTREM-1 had a higher correlation with SOFA scores, suggesting that sTREM-1 was more closely related to disease conditions than both CRP and PCT.

During the 14-day observation period, sTREM-1 levels in the non-surviving group increased gradually over time, whereas sTREM-1 levels tended to gradually decrease in the surviving group. This indicates that the inflammatory indicator sTREM-1 may be associated with sepsis prognosis. That is, a progressively decreasing expression of sTREM-1 indicates that the infection-induced inflammatory responses are being controlled and that a patient's prognosis is good.

sTREM-1 is primarily produced by the hydrolysis and shedding of membrane-bound TREM-1. Thus, progressive increases of sTREM-1 levels indicate that the total expression of TREM-1 continuously increases and that more pro-inflammatory cytokines and mediators are being released in the body. TREM-1 levels are further increased via positive feedback mechanisms, suggesting the persistence or progression of excessive inflammatory responses and poor prognoses.

Different from sTREM-1 levels, CRP and PCT levels in the surviving group were lower than those in the non-surviving group, indicating that patients with higher expressions of CRP and PCT may have poorer prognoses (although CRP and PCT levels tended to decrease in both groups over time). Overall, these results suggest that dynamic changes of sTREM-1 may better reflect the body's state of inflammatory response and sepsis severity simultaneously making sTREM-1 superior to CRP and PCT. Progressive increases in serum sTREM-1 levels is an indicator of a poor prognosis.

The changing trends for sTREM-1, CRP and PCT identified in the current study were consistent with those in previous studies [11,12]. Nonetheless, it remains debatable whether or not initial sTREM-1 levels are directly related to prognosis. Gibot et al. [11] found that initial sTREM-1 levels in a non-surviving group were lower than those in a surviving group, and suggested that prognosis would be poorer for patients with lower initial sTREM-1 levels. One possible reason for this finding is a hypothesis that sTREM-1 may compete with membrane-bound TREM-1 for ligand binding and thereby attenuate the transmission of infectious signals from membrane-bound TREM-1 into cells. Thus, the release of pro-inflammatory cytokines and mediators may be reduced and excessive inflammatory reactions and injury may be abrogated. Another possible reason is that inhibitory DAP12 receptors are present on the cell membrane that can bind with sTREM-1. DAP12 receptors negatively regulate TLR signalling pathways, thereby preventing excessive inflammatory responses. Thus, sTREM-1 may have certain anti-inflammatory and protective effects [13]. Patients with low levels of sTREM-1 are prone to excessive inflammatory responses and have poor prognoses.

Our results are consistent with those reported by Giamarellos-Bourboulis et al. [12] who reported that initial sTREM-1 levels in a non-surviving group were higher than those in a surviving group. There are several possible reasons for this observation. First, sTREM-1 is produced when membrane-bound TREM-1 is cleaved from the cell surface. High expression levels of sTREM-1 reflect the high expression of membrane-bound TREM-1. The initial disease conditions were more severe in the non-surviving group and systemic inflammatory reactions were obvious. The expression of membrane-bound TREM-1 increased and the inflammatory reaction caused injury to the body's cells and tissues resulting in the production of endogenous inflammatory pathogenic factors and an increase in the expression of TREM-1. As a result, the initial sTREM-1 levels in the non-surviving group were higher than those in the surviving group. Second, although sTREM-1 may have some anti-inflammatory effects, infections were not well controlled in the non-surviving group. As a result, inflammatory responses overwhelmed the body's compensatory anti-inflammatory capacities. This excessive inflammatory response was persistent resulting in a continuous high expression of membrane-bound TREM-1 and an increase in the total amount of sTREM-1 produced by its hydrolysis. Third, because some of our patients developed sepsis at other hospitals and were transferred to the ICU due to poor therapeutic effects, the observed initial sTREM-1 levels might not actually have been the levels at the onset of sepsis. Instead, the measured levels might actually have been at a later stage in the non-surviving group, which were higher than those measured in the surviving group. Finally, the small sample size for this study could also have influenced the results.

Although the relationship between initial sTREM-1 levels and prognosis remains controversial, multiple studies had similar findings showing that patients with progressively decreasing sTREM-1 levels had better prognoses, whereas patients with progressively increasing sTREM-1 levels had poorer prognoses.

## Conclusions

In summary, sTREM-1 levels can reflect sepsis severity more accurately than CRP and PCT and the dynamic changes of sTREM-1 are more sensitive for predicting prognosis. However, the sample size for this study was quite small and larger studies are needed.

## Competing interests

The authors declare that they have no competing interests.

## Authors' contributions

JZ designed the study, carried it out, performed the data analysis and wrote the first draft of the manuscript. DS and YJ conceived the initial idea for using sTREM-1 levels for infectious diseases and supplemented the study design. DF guided the data analysis and the use of medical statistics. LX was responsible for protocol revisions, data analysis and final draft revision. All authors read and approved the final manuscript.

## Pre-publication history

The pre-publication history for this paper can be accessed here:

http://www.biomedcentral.com/1471-2334/11/53/prepub
